# Latency of inhibitory response

**DOI:** 10.1186/1471-2202-14-S1-P235

**Published:** 2013-07-08

**Authors:** Marie Levakova, Petr Lansky

**Affiliations:** 1 Department of Mathematics and Statistics, Faculty of Science, Masaryk University, 611 37 Brno, Czech Republic; 2 Institute of Physiology, Academy of Sciences of the Czech Republic, 14220 Prague 4, Czech Republic

## 

Response latency in spiking activity is frequently investigated and often defined in different ways. In this study it is the time period from the stimulus application to the change in the neural firing rate evoked by the stimulation. While methods of latency estimation were proposed mostly for excitatory response (e.g. in [1], [2], [3]), the opposite kind of reaction has not been investigated. Therefore, we focus on the estimation of the latency in the case of inhibitory response.

The approach is based on observations of the time from the stimulus onset to the occurrence of the first spike after the stimulus (forward recurrence time) in *n *independent trials (the same approach as in [3]). Two types of models of a spike train are introduced. The characteristic feature of the first type is the assumption of a constant latency across trials. On the contrary, in models of the second type, the latency is a random variable; hence it can vary across trials. The statistical properties of the latency are estimated in the second case.

Either the probability density function of the forward recurrence time or its Laplace transform is derived for a given model and is then employed in several methods of latency estimation. Moment estimators and maximum-likelihood estimators are applications of generally known estimation methods. Another method uses the Laplace transform of the probability density function of the forward recurrence time. And the last proposed method is semiparametric and its idea is to compare the theoretical cumulative distribution function of the forward recurrence time derived for the model in absence of stimulation to the empirical distribution function obtained from data.
